# “I don’t Think These Devices are Very Culturally Sensitive.”—Impact of Automated Speech Recognition Errors on African Americans

**DOI:** 10.3389/frai.2021.725911

**Published:** 2021-11-26

**Authors:** Zion Mengesha, Courtney Heldreth, Michal Lahav, Juliana Sublewski, Elyse Tuennerman

**Affiliations:** ^1^ Department of Linguistics, Stanford University, Stanford, CA, United States; ^2^ Google Research, Google, Seattle, WA, United States; ^3^ dScout, Chicago, IL, United States

**Keywords:** fair machine learning, natural language processing, speech to text, African American Vernacular English, sociolinguistics, social psychology, artificial intelligence

## Abstract

Automated speech recognition (ASR) converts language into text and is used across a variety of applications to assist us in everyday life, from powering virtual assistants, natural language conversations, to enabling dictation services. While recent work suggests that there are racial disparities in the performance of ASR systems for speakers of African American Vernacular English, little is known about the psychological and experiential effects of these failures paper provides a detailed examination of the behavioral and psychological consequences of ASR voice errors and the difficulty African American users have with getting their intents recognized. The results demonstrate that ASR failures have a negative, detrimental impact on African American users. Specifically, African Americans feel othered when using technology powered by ASR—errors surface thoughts about identity, namely about race and geographic location—leaving them feeling that the technology was not made for them. As a result, African Americans accommodate their speech to have better success with the technology. We incorporate the insights and lessons learned from sociolinguistics in our suggestions for linguistically responsive ways to build more inclusive voice systems that consider African American users’ needs, attitudes, and speech patterns. Our findings suggest that the use of a diary study can enable researchers to best understand the experiences and needs of communities who are often misunderstood by ASR. We argue this methodological framework could enable researchers who are concerned with fairness in AI to better capture the needs of all speakers who are traditionally misheard by voice-activated, artificially intelligent (voice-AI) digital systems.

## Introduction

With the advances in deep learning for speech, and natural speech and language processing, ASR systems have improved dramatically over the past several years and have become ubiquitous in everyday life. Examples of ASR include virtual assistants, automatic translation, digital dictation, and hands-free computing. Given the rise of popularity of these voice-based systems, failures of ASR systems can pose serious risks to users. For example, in crisis management situations, poor quality of speech input can pose real challenges for speech recognition systems (Vetulani et al., 2010). In the health context, being misunderstood by ASR systems can lead to patient harm (Topaz et al., 2018). Therefore, the importance of being understood by speech recognition (and the consequences of being misunderstood) requires a closer investigation.

There is growing evidence ASR systems exhibit racial bias (Koenecke et al., 2020), which is a problem that has become more apparent in many other areas of machine learning such as face recognition (Buolamwini and Gebru, 2018), healthcare (Obermeyer, powers, Obermeyer et al., 2019), natural language processing (Blodgett et al., 2016; Su et al., 2016), and online advertising (Ali et al., 2019). While there is concern that these systems do not work equally well for everyone (Harwell et al., 2018; Tatman, 2017), the methods ASR researchers use to address this inequality have mostly been at odds with their motivations. Blodgett and others (2020), for example conducted a survey of 146 papers which analyzed bias in natural language processing (NLP) systems found that “quantitative techniques for measuring or mitigating ‘bias’ are poorly matched to their motivations and do not engage with the relevant literature outside of NLP” (Blodgett et al., 2020, p. 1), illuminating the need for speech recognition researchers to look to literature in fields where the relationship between language and social stratification has been established, such as sociolinguistics, linguistic anthropology, social psychology, and sociology.

Studying African Americans’ speech in sociolinguistics has long revealed the importance of understanding the relationship between bias toward visual bias African Americans and bias toward African Americans’ speech, or African American Vernacular English (AAVE). While there are different ways of defining, and indeed different names for the dialect, we adopt King, 2020 definition of AAVE which is the any language spoken by African Americans, while also acknowledging that AAVE is a systematic, rule-governed language of African American descent communities (Rickford, 1999a). The landmark case of Martin Luther King Junior Elementary School Children v. Ann Arbor School District (1979) first brought to public awareness the harmful effects of dialect discrimination toward AAVE. Eleven African American children were wrongly placed in special education when Language Arts teachers and standardized tests failed to capture their command of English. The Michigan Supreme Court ruled that the school’s failure to recognize AAVE violated federal law and ordered the district to design teaching methods that considered the grammar and phonology of AAVE. While this represents one advance toward linguistic equality, further research has unearthed the systematic nature of dialect discrimination toward AAVE in classrooms (Williams et al., 1971; Rickford, 1999b), courtrooms (Rickford & King, 2016; Jones et al., 2019), hospitals (Nelson, 2002), housing discrimination (Purnell et al., 1999; Baugh, 2003), and employment discrimination (Henderson, 2001; Grogger, 2011). However, research in speech systems, which necessarily interfaces with speakers spanning a range of dialectal backgrounds (including AAVE), rarely consider this extensive body of sociolinguistic and dialect discrimination research. There is a gap in our understanding of how the insights, concepts and methods from sociolinguistics and social psychology ought to inform ASR research which this study aims to fill.

Thus far, limited work has incorporated sociolinguistic theory into ASR fairness. In their groundbreaking study of the five largest providers of speech technology, e.g., Amazon, Apple, Google, IBM, and Microsoft, Koenecke et al., 2020 found that African Americans experience word error rates up to two times higher than White, standard American English speakers. These performance gaps revealed a new category of dialect discrimination, finding that speech models are disproportionately confused by the phonetics, phonology, and prosody of AAVE. While other studies have explored the ASR failures via the word error rate (WER) for African American speakers, no studies to date have explored the experiential effects of these failures. Furthermore, an unresolved question remains: what are the psychological effects of being misheard by voice technology on African Americans? In the present study, we use the diary method to capture African American users’ experiences and needs in real time and address this limitation. By utilizing a diary study, we contribute to a novel understanding of experiences with ASR systems. More specifically, no research to date has explored both day-to-day and infrequent (yet impactful) experiences that contribute to a user’s perceptions of ASR systems. In this work, we take a step toward investigating the daily experiences that affect African American users’ perceptions of and behaviors with ASR systems. To achieve this, we conducted a diary study of 30 African American users of ASR and asked participants to report their experiences -- specifically the salient moments when they felt frustrated with voice technology -- over a 2-week period. To place these frustrations in the context of participants’ broader experiences with ASR systems, we also asked participants to describe an experience where ASR systems did not work for them in the past.

Researchers have only just begun to recognize the need to bridge the gap between bias in NLP systems and literature outside of NLP, particularly “a greater recognition of the relationships between language and social hierarchies, encouraging researchers and practitioners to articulate their conceptualizations of ‘bias’ -- i.e., what kinds of system behaviors are harmful, in what ways, to whom, and why (…) -- and to center work around the lived experiences of members of communities affected by NLP systems, while interrogating and reimagining the power relations between technologists and such communities” (Blodgett et al., 2020). Thus, a greater understanding of the daily perceptions and experiences of African American users of voice technology can help the speech recognition community address many of the challenges African American users face when using voice technology.

## Methods and Participants

### Recruitment

Participants were recruited on the dScout^1^ platform using a screening survey. In the screening survey, respondents (*n* = 1,865) were asked how frequently they used voice-technology; how often errors occur when using voice-technology; the reasons why they believe these errors occur and a series of demographic questions about gender, race, age, income, and level of educational attainment.

### Participants

Among the survey respondents, 30 African American participants, all native English speakers, completed the diary study, and were paid $150 for their participation. dScout participants all lived in or near the city of Atlanta, Chicago, Houston, Los Angeles, New Orleans, Philadelphia, or Washington D. C, and were balanced for age, gender, income, and education-level^2^. Additionally, participants used voice technology in at least one Google product, reported using voice technology a few times a month or more, reported experiencing errors with voice technology, and, crucially, reported that they believe errors with voice technology occur due to the way they speak.

### Methodology

The primary aim of our research was to understand the impact of errors on African Americans who use voice technology. Because we wanted to understand daily perceptions and experiences of voice technology, we chose a diary study method in which participants captured each experience of ASR failure in their own time, without prompting by the researchers (Carter and Mankoff, 2005). Participants submitted videos, as well as supplementary closed-ended and open-ended questions, to a mobile diary through the dScout smartphone application.

### Protocol

The study took place over 2 weeks and consisted of five activities. These activities included both single-submission videos and diary-style question sets, sequenced such that participants shared all their in-context diary reflections before moving on to the survey-style assignments. The survey and activities were distributed as five distinct parts, and a data-usage consent was also provided.

In the first part, which lasted 1 day, we asked participants to share an overview of what works well and does not work well when using voice technology, the emotions that they feel when using voice technology, and their overall level of satisfaction. Participants were also asked about voice modification, e.g.,: “Have you ever modified the way you talk to get different results when using voice technology?” For those who indicated that they modify their speech, we asked an open-ended question about this experience in which the participant was able to report any emotions they feel as a result of needing to modify their speech. To conclude part 1, participants were asked several questions to understand the role that speaker-identity played in speech-modification.

The second part consisted of a diary-style survey completed over the course of 5 days. Participants were instructed to report any moment when they used voice technology for any purpose, including both dictating text and giving a voice command, describing the activity they were completing with voice technology and their intent in choosing voice technology for the task, indicating which service(s) and/or device(s) were used, and whether anyone else was involved in using the voice technology in the moment. Participants also answered a specific set of questions in-the-moment when using voice technology and repeated this at least three times. Lastly, participants’ level of satisfaction with each voice-tech interaction was obtained through both a close-ended question and a 1-min video.

The third and fourth parts of the study focused on users’ negative experiences with voice technology and, collectively, lasted 2 days. In part 3, participants were asked to describe any instance in which their experience with voice technology was “bad” or “negative” through open and closed-ended questions. In part 4, participants were asked to recreate their negative experience through a screen recording or video capture.

In the fifth part, participants were randomly assigned to complete one of seven specific voice technology activities — 1) sending a message to a friend or family member 2) creating a reminder, 3) writing an email to a work colleague, 4) getting information to address an important personal issue, 5) completing a Google search, 6) getting directions to a nearby grocery store, or 7) calling a close friend or family member. Participants were asked to reflect on their assigned experience and rate their level of satisfaction with the voice technology while completing the activity. To conclude part 5, participants responded to a series of Likert questions related to their perception of how the voice technology interpreted their prompt (Appendix). Participants had 1 day to complete this task and a final reflection.

### Analysis

To report experiences, participants recorded 60-s videos and answered closed and open-answer survey questions, when applicable, about experience, perception, and feelings they had about voice technology. The videos were human transcribed by the researchers conducting analysis on the study, word by word as they were being watched.

Six hundred forty unique open-ended responses, 1,080 closed-ended responses, and 240 video transcripts were analyzed. Open-ended response and video transcript data were comprehensively and separately analyzed with a unique set of thematic codes being applied to each set of distinct question responses. 124 individual codes were developed (Appendix B).

Thematic codes were developed through a bottom-up analysis approach. This approach entailed one researcher reading 100% of written and transcript responses to a singular question to identify a set of up to 11 repeat patterns within the responses to each question. As patterns were identified, the researcher kept track of how often they were mentioned. Patterns that were mentioned in two or more responses to that singular question were turned into concise codes that captured the sentiment and type of response provided. To communicate the prevalence of each code, the researcher developed a document of the code set for each individual question, organizing the codes from most frequently mentioned to least frequently mentioned.

## Results

### ASR Failures With African American Vernacular English

**FIGURE 1:**
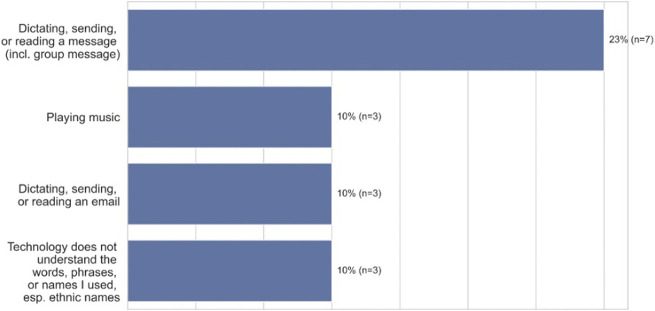
Lack of Satisfaction of ASR Technology among African-American participants (*n* = 30).

The reasons voice technology failed varied. As Figure 1 (open-ended question responses) shows, participants reported that voice technology did not work well when sending, replying to, or dictating a message (23%, *n* = 7), playing music (10%, *n* = 3), with specific names (10%, *n* = 3), or dictating, sending, or reading emails (10%, *n* = 3). As an example, P7, a 24–26 -years old in Chicago, Illinois reported that when using Siri to make a phone call, “*It called the wrong person, and I ended up having an awkward moment with someone and it felt super weird*.” Additionally, the system did not seek confirmation of the person’s name. In order to improve the experience, the participant said, “*I would have had it audibly say who it was calling as a final double check*.” However, in cases such as this, participants felt they needed to execute their tasks manually. For example, users reported going to the timer app themself to set a timer, rather than asking Siri to set the timer via voice. As P19, a 51–53 years old woman from the Bronx, New York noted, “*I might as well have typed it out myself instead of just going back again rereading every word, deleting words, and adding words.*”

**FIGURE 2:**
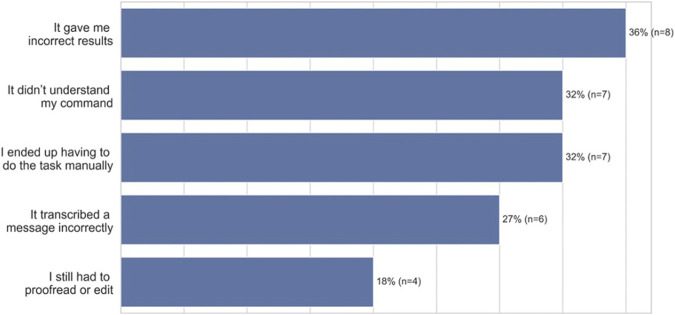
Why African American participants aren’t satisfied (*n* = 22).

Participants expressed dissatisfaction when voice technology made it difficult for them to achieve their goals. For example, participants felt dissatisfaction when voice technology gave them incorrect results as a product of being misheard (36%, *n* = 8), or when it didn’t understand a command (32%, *n* = 7). In addition, participants felt dissatisfaction when they ended up having to do the task manually (32%, *n* = 7), if the technology mistranscribed a message (27%, *n* = 6), or if they still had to proofread or edit (18%, *n* = 4) (Figure 2, open-ended question responses). As an example, P9, a 25–27 years old from Peoria, Illinois reported, *“It conveyed the opposite message than what I had originally intended, and cost somebody else a lot (of time).”*

### Psychological Impact of ASR Failures on African Americans

**FIGURE 3:**
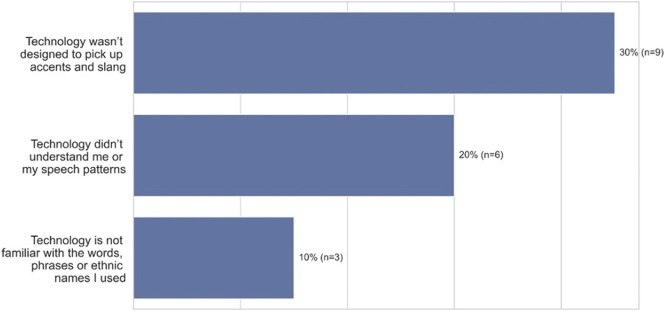
Top three attribution of errors among African-American participants when using ASR technology (*n* = 30).

One of the main goals of this research was to understand the psychological impact of voice errors on speakers of African American Vernacular English (AAVE), and in several cases, participants expressed that voice technology did not work because the system didn’t understand the way that they spoke. As Figure 3 (open-ended question responses) shows, 30% (*n* = 9) of participants mentioned that errors occurred because the technology wasn’t designed to comprehend accents or slang. In addition, participants reported that failures occur because the technology doesn’t understand their speech patterns (20%, *n* = 6), or the words, phrases, and names they use (10%, *n* = 3). For example, a 24–26 years old from Naperville, Illinois stated, *“I’ve had to repeat certain words because they did not understand the vernacular I have.”* In discussing African American names, P12, a 47–49 years old from South River, New Jersey states, *“I think the spelling or pronunciation of ethnic names played a part in the unsuccessful result. I think that the programmers input (only) common non-ethnic names in the programming.”*

**FIGURE 4:**
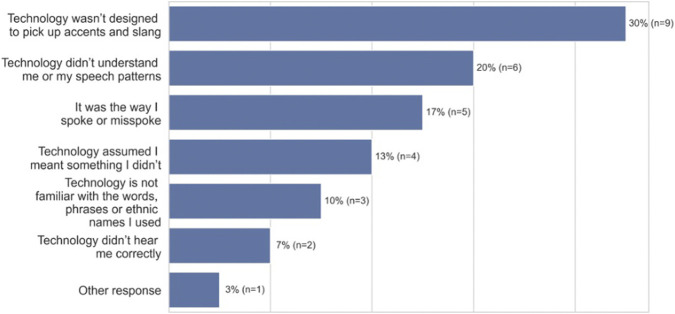
Attribution of errors among African-American participants (*n* = 30).

When participants were asked whom they attribute the errors and failures to, most said they attribute the errors to the technology. As shown in Figure 4 (open-ended question responses), they stated, in order of magnitude, “*the technology wasn’t designed to pick up accents and slang*” (30%, *n* = 9), “*the technology didn’t/doesn’t understand me or my speech patterns, or natural speaking patterns*” (20%, *n* = 6), “*the technology assumed I meant something I didn’t*” (13%, *n* = 4)*,* “*the technology is not programmed to understand the words, phrases or ethnic names I used*” (10%, *n* = 3), “*the technology didn’t hear me correctly* (7%, *n* = 2)*.*” However, some African Americans attributed the errors to themselves, stating “*it was the way I spoke or misspoke (including too fast).*” (17%, *n* = 5)*.* In other words, some are placing attribution on themselves for deficiencies and biases in the technology.

**FIGURE 5:**
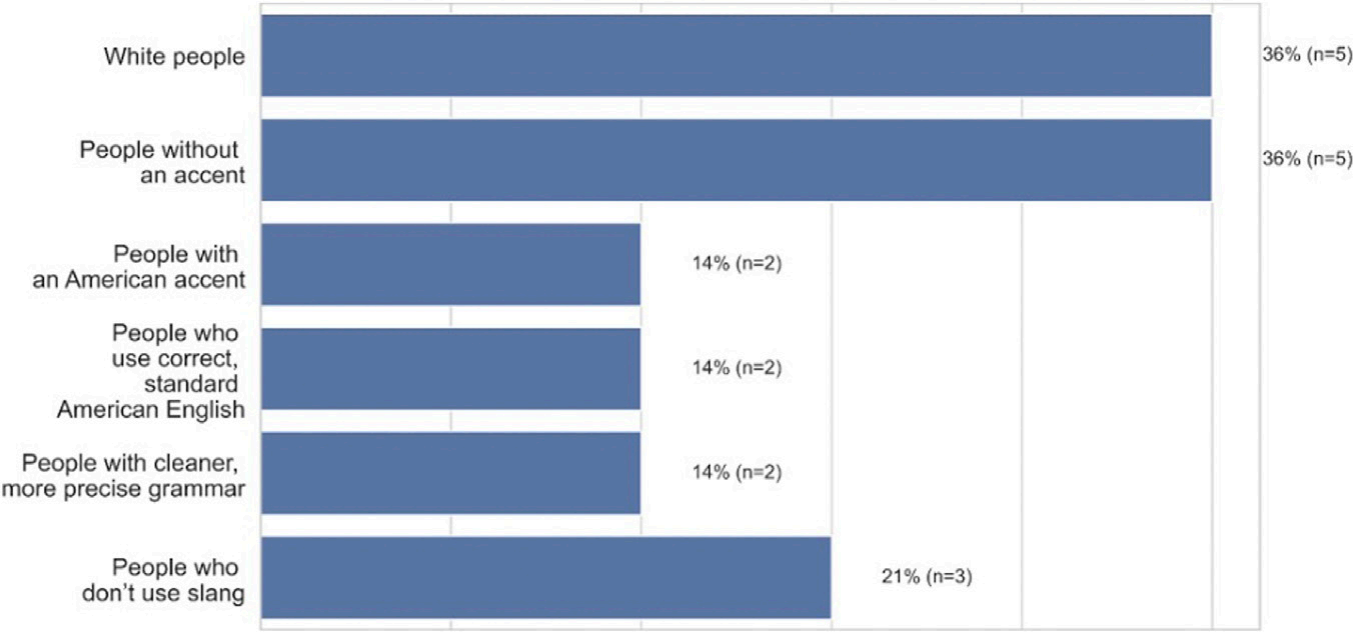
Who African-American participants believe ASR technology works better for (*n* = 14).

When asked for whom the technology works better for in an open-ended response, 36% (*n* = 5) of participants reported “white people”, 36% (*n* = 5) reported people without an accent, while 14% (n = 2) indicated that the technology works better for people with an American accent, people who use correct, standard American English (14%, *n* = 2), or people with cleaner or more precise grammar (14%, *n* = 2) (Figure 5, open-ended question responses). Taken together, a majority of African Americans think that ASR works best for White speakers or standard English speakers. As P7, a 25–27 years old in Chicago, Illinois states “*The technology is made for the standard middle-aged white American, which I am not*.”

**FIGURE 6:**
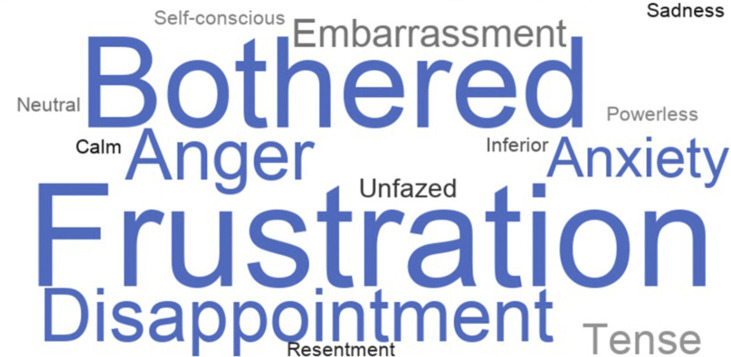
Emotions experienced from Voice Technology Errors (*n* = 30).

**FIGURE 7:**
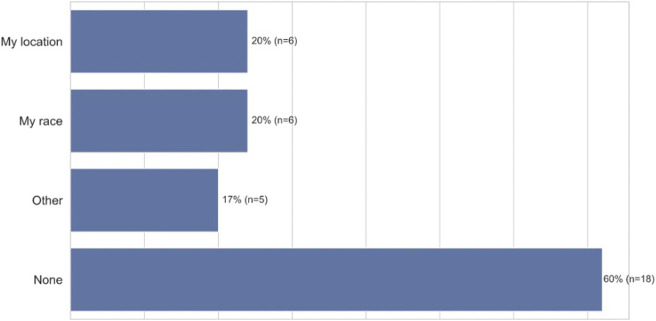
Attributes considered when African-American participants encountered ASR technology errors (*n* = 30).

These voice errors had psychological and emotional consequences for our African American participants. When voice errors occur, a majority of participants experienced frustration (77%, *n* = 23), felt bothered (58%, *n* = 17), disappointed (55%, *n* = 16), and angry (52%, *n* = 15). Some participants even experienced anxiety from ASR failures (36%, *n* = 10) (Figure 6, closed-ended question responses). Furthermore, we found that voice errors activate certain aspects of participants’ identities. When we asked participants which personal attributes came to mind when ASR failures occurred some participants indicated that they thought their racial identity was a factor (20%, *n* = 6), while others thought their regional and location identity was a factor (20%, *n* = 6) (Figure 7, closed-ended question responses). As P3, a 47–49 years old from Chicago, Illinois, states “*I was thinking that because of my slightly ethnic tone I feel and it was hard for the talk-to-text to clearly understand what I was stating*.” In some instances, both thoughts of race and location surfaced when voice errors occurred. This was illustrated by P5 a 18–20 years old from Garland, Texas when she states, “*Because of my race and location, I tend to speak in a certain way that some voice technology may not comprehend. When I don’t speak in my certain dialect, I come to find out that there is a different result in using voice technology*.”

### Behavioral Impact of ASR Failures on African Americans

**FIGURE 8:**
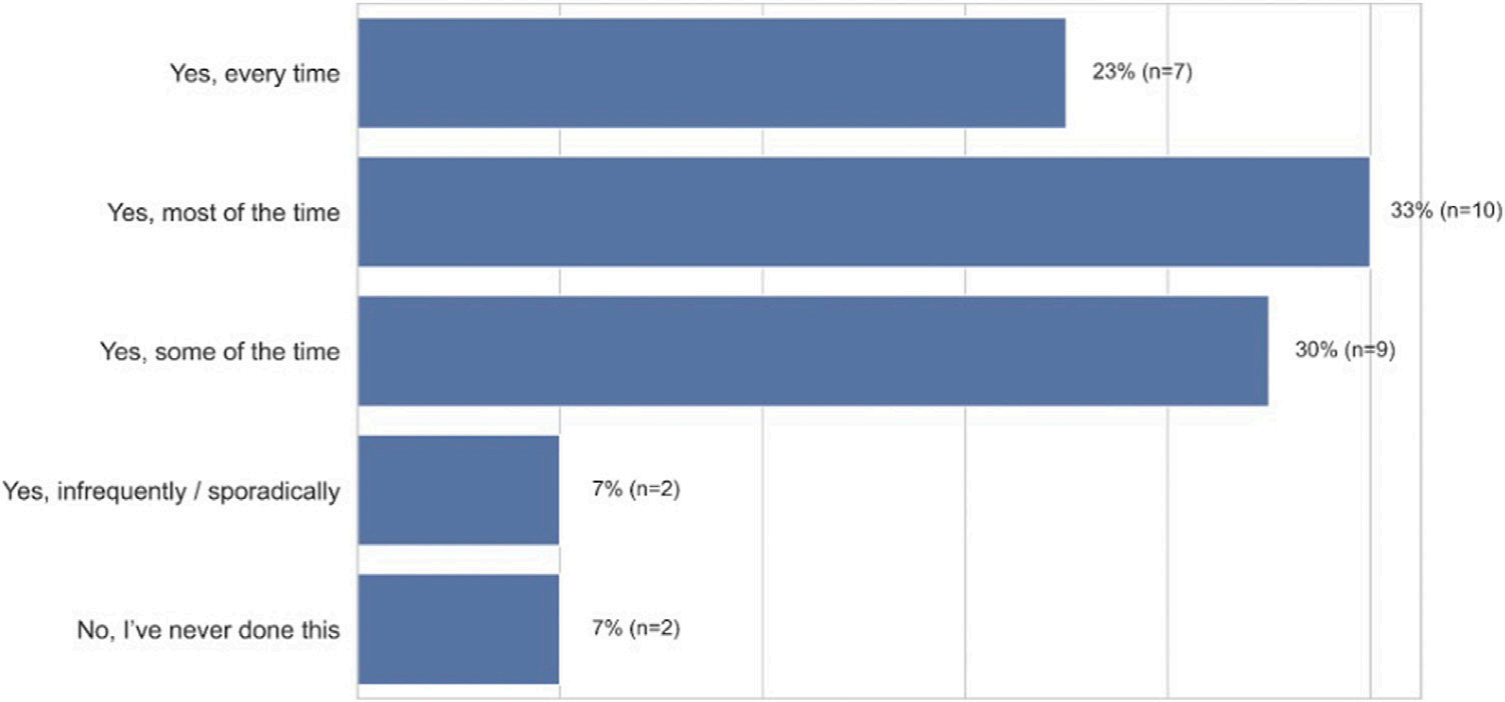
Frequency of speech modification by African-American participants when using ASR technology (*n* = 30).

African American users reported having to work around the aforementioned issues by accommodating their speech to meet the limits of voice technology, as ASR produces twice the WER for African American speakers as compared to White speakers (Koenecke et al., 2020). Linguistic accommodation is a form of speech modification, where people alter their phonemes, word choice, and syntax to meet the expectations about standardness based on the situation or person one is talking to (Giles & Coupland, 1991). As Figure 8 (closed-ended question responses) shows, most participants reported modifying their dialect in order to be comprehended by voice technology (93%, *n* = 28). Most participants reported having to accommodate the way they spoke in order to be understood. P7, a 24–26 years old in Chicago, Illinois, illustrates this need to accommodate when he states: “*I modify the way I talk to get a clear and concise response. I feel at times, voice recognition isn’t programmed to understand people when they’re not speaking in a certain way*.” In addition, P8 also talks about the process of altering his language when he states, “*What usually works for me is when I talk real clear, and don’t use slang words like my regular talk*.”

**FIGURE 9:**
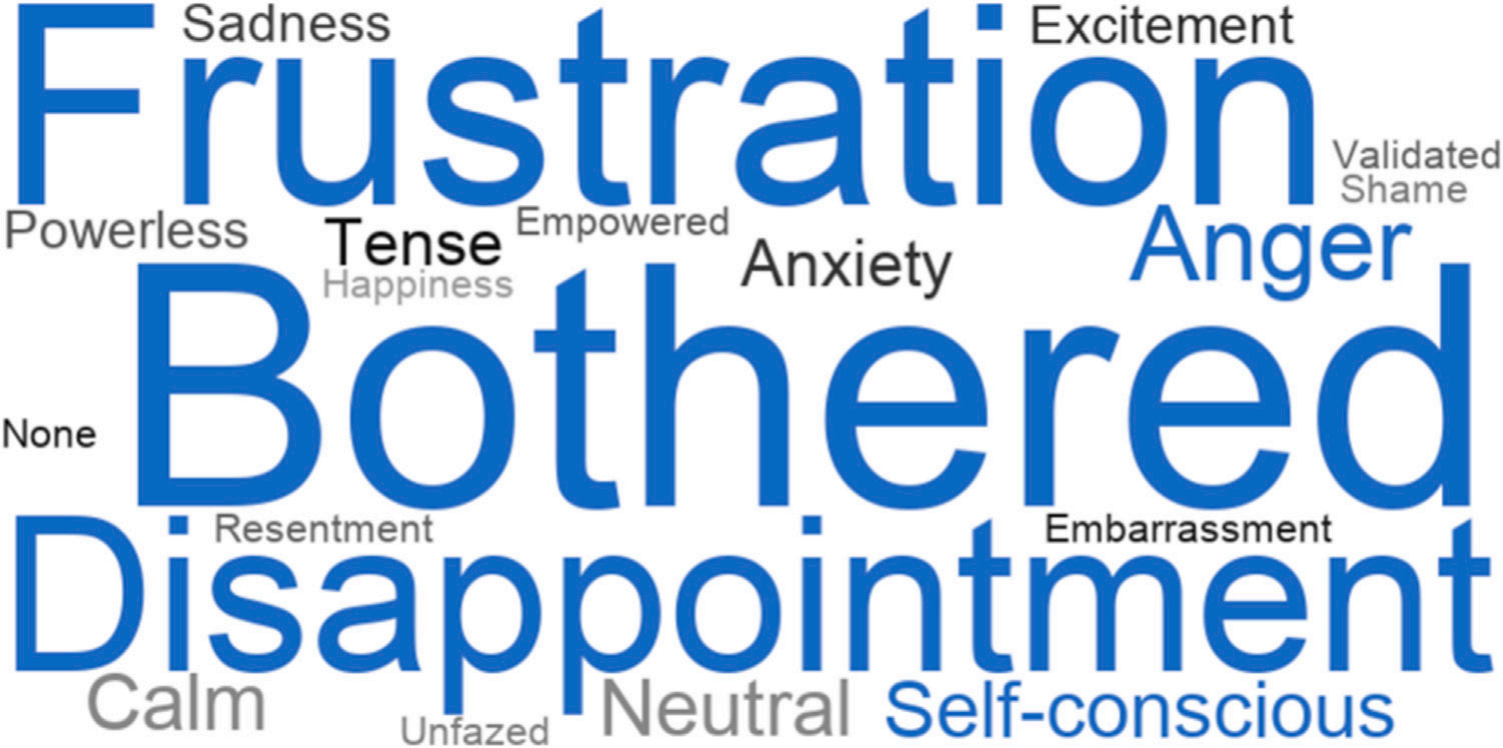
Emotion Experienced when participants accomodated for Voice Technology Errors (*n* = 30).

**FIGURE 10:**
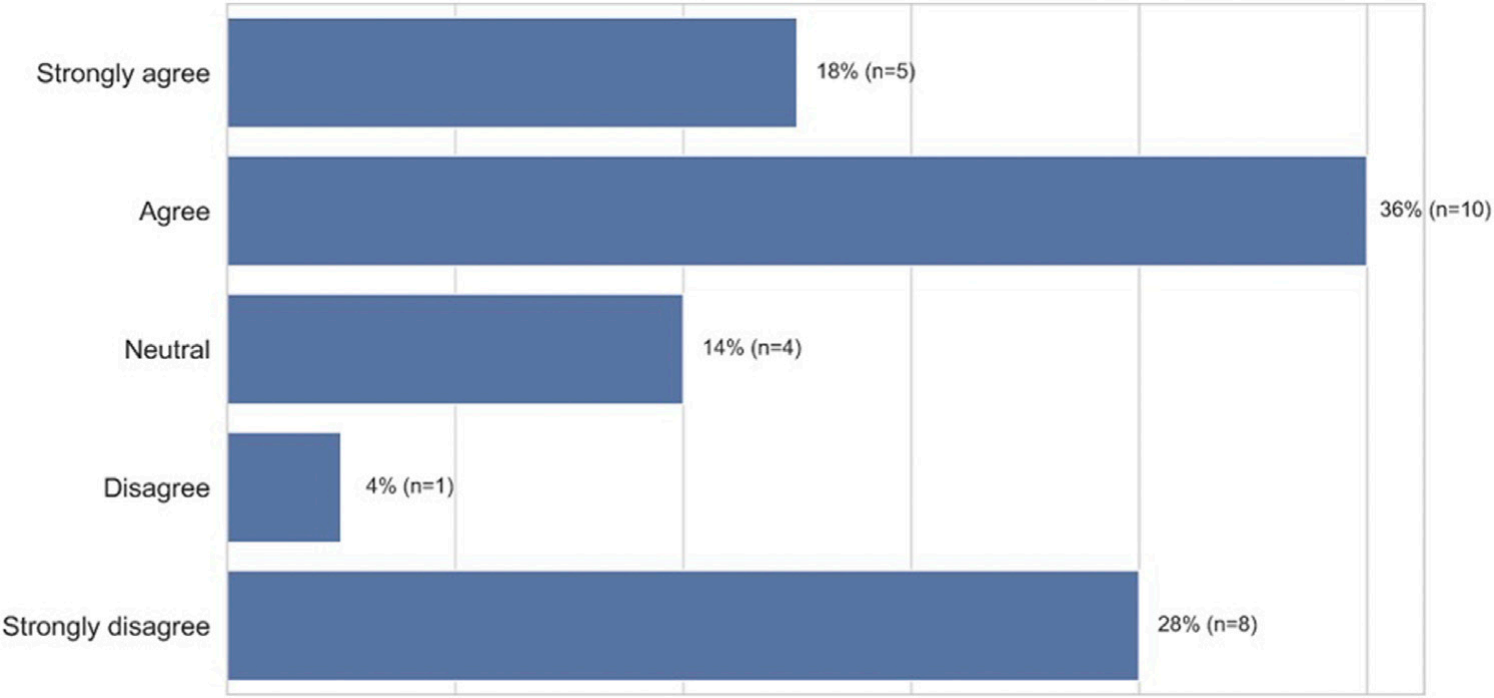
African-American participants needed to modify their speech for different results (*n* = 28).

We found that the act of accommodation triggered a variety of negative emotional responses, the top five being bothered (67%, *n* = 20), frustration (53%, *n* = 16), disappointment (40%, n = 12), anger (33%, n = 10), and self-consciousness (17%, *n* = 5) (Figure 9, closed-ended question responses). As before, participants perceived the need to accommodate as an artifact of being outside the group the technology was built for. When we explicitly asked participants whether they needed to modify the way they talk to get different results when using voice technology because the technology doesn’t understand people who come from their racial group, 54% (*n* = 15) “strongly agreed” or “agreed” with this statement. More granularly, 18% (*n* = 5) “strongly agreed” and 36% (*n* = 10) “agreed” while 14% (*n* = 4) were “neutral,” 4% (*n* = 1) “disagreed,” and 28% (*n* = 8) “strongly disagreed” with the statement (Figure 10, closed-ended question responses). For example, P12, a 47–49 years old from South River, New Jersey, specifically points to technology companies’ lack of internal diversity being the cause of non-inclusive voice systems: “*I think the spelling or pronunciation of ethnic names played a part in the unsuccessful result. I think that the programmers’ input (only) common non-ethnic names in the programming or they don’t employ people from multicultural backgrounds to get a wider range of speech and voice inflections.*” Furthermore, participants like P14, a 18–20 years-old from Baltimore, MD, explicitly states that she would have to change who she is—not simply her dialect—for the technology to work for her: “*It [voice technology] needs to change because it doesn’t feel inclusive when I have to change how I speak and who I am, just to talk to technology*.”

## Discussion

As we go through our daily lives, we experience virtual assistants, automatic translators, digital dictation, and hands-free computing powered by ASR systems that are rarely free of the effects of bias. With a growing concern that these systems exhibit bias toward African American Vernacular English, this study investigated African Americans’ experiences with ASR failures. Longitudinal and cross-sectional research has produced a large body of evidence demonstrating the robust association between discriminatory experiences and negative psychological well-being (e.g., Brody et al., 2006; Seaton et al., 2008). The effects of these ASR failures suggest that African Americans who use these systems experience negative emotions when errors occur, and these emotions are consistent with emotions that are reported when individuals feel discriminated against, such as frustration, anger, and anxiety (Carter and Forsyth, 2010; Clark et al., 1999; Harrell, 2000). These findings are relevant because emotions play an important role with coping with racial discrimination. Indeed, we found that African Americans feel othered when using technology powered by ASR—errors surface thoughts about identity, namely about race—leaving users feeling that the technology was not made for them. This was substantiated by personal accounts. For example, P11, a participant from Los Angeles, CA noted, “*I don’t think these devices are very culturally sensitive* (*…*) *it often doesn’t understand what you’re saying, because it doesn’t understand dialects of different people, in any way*.”

African Americans reported accommodating their speech to be understood by their technology, suggesting that they are adapting some features of African American Vernacular English in order to get more successful results. Speech accommodation is an adaptation of one’s syntax, phonetics, phonology, prosody, or other fine-grained phonetic details in order to talk more like their conversation partner (Giles, 1979). Currently, most American English voice-AI systems are programmed with standard American English voices, though this is beginning to change (Waddell, 2021). While there is limited work on the linguistic properties of speech accommodation to voice-AI, Cohn et al., 2019 found that, following an ASR error, speakers adapt their original utterance by lengthening the duration of their vowels, and it should be noted that vowel length duration is not a regional dialectal feature. Future research should explore how African American speakers are adapting their speech in order to be understood by voice-AI, and whether this adaptation involves changing features of their dialect. This will allow for the documentation of which phonetic or phonological features of AAVE are most commonly misunderstood by ASR systems and inform the collection of relevant speech samples to improve errors.

### Suggested Actions for Mitigating Racial Biases

If errors surface negative thoughts and emotions, what can we, as linguists and speech recognition researchers, do to mitigate the psychological effects of ASR failures for African Americans and the millions of non-standard dialect speakers? We end with some specific suggestions for what we can do.

**FIGURE 11:**
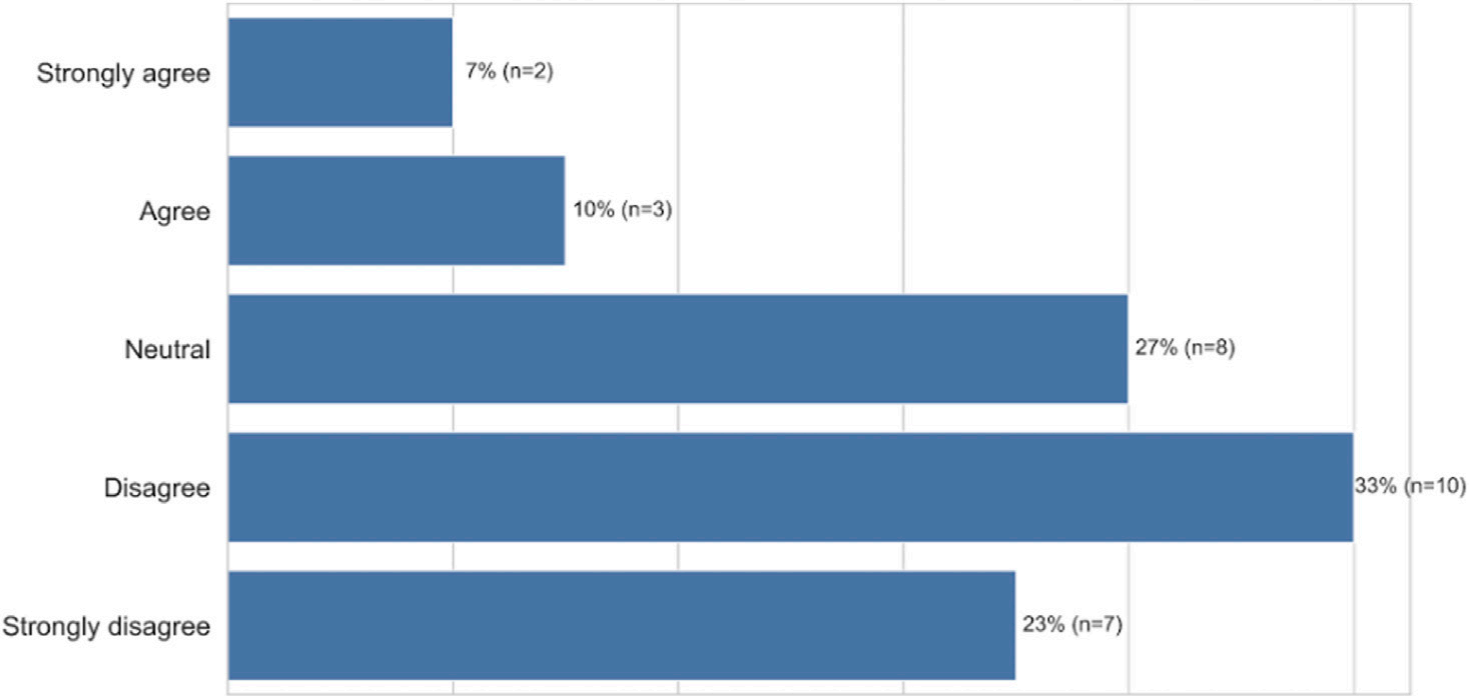
African-American participants belief ASR technology will not improve for users like themselves (*n* = 30).

1) Expand and diversify our data sets. Firstly, this study’s findings suggest the need to develop more diverse training datasets and models that include not only African American Vernacular English, but other underrepresented accents and vernaculars. Interestingly, participants expressed willingness to contribute to that change (Figure 11). As P14, a 18–20 years old from Baltimore, MD noted in her diary sample, she would be “*willing to share things such as my voice samples to show voice technology companies how vernacular is different and that everyone cannot speak robotically to a piece of technology*.”

Although users reported willingness to contribute personal information, such as voice samples and geographic location to improve the experience for themselves and their community, it is vital we explore this in a mindful and sensitive way, ensuring we protect user privacy and anonymity. Furthermore, there is an assumption that algorithmic biases can be mitigated by adding more data from different groups. But further research must be done to ensure that this increase in data represents individuals across socioeconomic status and access to devices. Hence, we also suggest future research includes the insights and lessons learned from work in sociolinguistics to build more inclusive voice systems that consider the heterogeneity of African American Vernacular English (King, 2020) across regions, genders, ages, and socioeconomic classes.

2) Personalized speech. Explore integrating personalized speech models that are trained to recognize users’ individual speech patterns, as well as providing clear pathways for error correction and federated repair.
3) Understand dialectical transcription preferences. Here we propose an opportunity to understand what African American users’ preferences are regarding the speech-to-text output of African American Vernacular English, including how users want their speech transcribed.
4) Involve community voices in the solution**.** Most importantly, we believe that involving the voices and perspectives of African American community members early and often in the product development cycle through community-based participatory research (CBPR) can address many of the challenges African American users’ face when using voice technology. A CBPR approach seeks to encourage impactful conversations on current issues and lived experiences with the goal of prioritizing and promoting social unity among historically marginalized communities (Barnidge et al., 2010; Coughlin and Smith, 2017; Vaughn et al., 2017). While CBPR has been applied widely in public health and related disciplines, we believe that this approach can enable a deeper understanding of the processes necessary for the success of ASR interventions by bringing African American voices and perspectives to the forefront to address these observed inequities.

Finally, while we believe one of the strengths of our research was to focus our study on a group susceptible to ASR failures (African-Americans), we recognize that experiences with voice technology could be poor for people irrespective of race and ethnicity. For example, contact dialing issues might occur at similar rates for AAVE and non-AAVE speakers, which does not necessarily point to an inclusivity problem. However, our results suggest that African Americans attribute errors to their race rather than the system, which suggests ASR systems are perceived to not work equally well for all subgroups and are not broadly inclusive. Future research should explore multi-ethnic experiences with ASR to understand how error attributions and linguistic accommodation vary across different races and ethnicities.

## Conclusion

There is a growing need for research on fairness in voice-AI to center its solutions around the lived experiences of members of communities underserved by voice-AI. Our findings suggest that the use of a diary study enables researchers to best understand the experiences and needs of communities who are often misunderstood by ASR. We argue this methodological framework could enable researchers who are concerned with fairness in AI to better capture the needs of all speakers who are traditionally misheard by voice-AI systems.

## Data Availability

The datasets presented in this article are not readily available because the datasets for this study are considered confidential and proprietary of Google. Requests to access the datasets should be directed to zmengesh@stanford.edu.

## Footnotes

1 dScout is a commonly used tool for conducting diary studies through participants’ mobile phones (Winnick, 2012). dScout maintains a panel of potential participants, who dScout recruits through a variety of methods (e.g., online advertisements, advertisements through frequent flyer programs, mailers, etc.) and is designed to ensure panel diversity -similar to non-probabilistic survey panels maintained by other companies such as Qualtrics (Redmiles et al., 2017).

2 While our selected participants represent a demographically diverse sample, there is a membership bias inherent in recruiting participants through a remote platform such as dScout. Individuals in the dScout panel must have access to a device running a recent version of the iOS or Android mobile operating system. Accordingly, the panel participants tend to be more technically-savvy, affluent, and educated than the general population. In terms of geographic distribution, these participants tend to reside in more densely populated urban areas as opposed to suburban or rural areas.

## Appendix:

Recruiting and research design questions (Appendix A) and thematic codes (Appendix B) can be found in the impact-of-errors-in-ASR repository here: https://github.com/elysetuennerman/impact-of-errors-in-ASR https://github.com/elysetuennerman/impact-of-errors-in-ASR
